# Plasma sphingomyelins increase in pre-diabetic Korean men with abdominal obesity

**DOI:** 10.1371/journal.pone.0213285

**Published:** 2019-03-05

**Authors:** Seung-Soon Im, Hyeon Young Park, Jong Cheol Shon, In-Sung Chung, Ho Chan Cho, Kwang-Hyeon Liu, Dae-Kyu Song

**Affiliations:** 1 Department of Physiology and Obesity-mediated Disease Research Center, Keimyung University School of Medicine, Daegu, Korea; 2 BK21 Plus KNU Multi-Omics Based Creative Drug Research Team, College of Pharmacy and Research Institute of Pharmaceutical Sciences, Kyungpook National University, Daegu, Korea; 3 Department of Occupational & Environmental Medicine, Keimyung University School of Medicine, Daegu, Korea; 4 Department of Clinical Endocrinology, Keimyung University School of Medicine, Daegu, Korea; Korea University, REPUBLIC OF KOREA

## Abstract

Abdominal or visceral obesity is a well-known risk factor for metabolic diseases. However, whether abdominal obesity significantly affects plasma lipid profile during the development of type 2 diabetes has not been fully elucidated. We investigated the differences in plasma lipid concentrations in 63 participants categorized into six groups (middle-aged Korean men); Normal, Pre-diabetes (pre-DM), and Diabetes mellitus (DM) with or without abdominal obesity (AO or lean). The lipidomic profiles were determined by using liquid chromatography-tandem mass spectrometry (LC-MS/MS). Sphingomyelin (SM) levels in plasma were significantly higher in the pre-DM with AO than in pre-DM with lean (*p* = 0.021). SM concentrations correlated positively with waist-to-hip ratio (WHR) (*r* = 0.256, *p* = 0.044), cholesteryl ester (CE) (*r* = 0.483, *p* < 0.0001), ceramide (*r* = 0.489, *p* < 0.0001) and plasmanyl phosphatidylcholine (PC) (*r* = 0.446, *p* < 0.0001). The present study found that pre-diabetic patients with AO were characterized by increased plasma concentrations of SM. Plasma SM levels in individuals with AO may be an early prognostic biomarker to better predict the progression toward type 2 diabetes and metabolic syndrome.

## Introduction

Obesity and type 2 diabetes are rapidly emerging as major public health problems worldwide. The rates of diabetes and obesity tended to increase in Korea [1]. Since all obese individuals are not a diabetic, the mechanism of linking obesity to diabetes is not clear yet. Indeed, Asians and Asian Americans have a lower prevalence of obesity as measured by body mass index (BMI) compared with Caucasians, but have a higher percentage of body fat at the same BMI as Caucasians [2]. In 2016, the Korean Diabetes Association reported that 58% of diabetic patients presented abdominal obesity (AO) and a quarter of the adults 30 years or older had pre-diabetes defined as impaired fasting glucose in the 100–125 mg/dL range [3]. Thus, the influence of AO on the risk of diabetes was found to be more important than that of general obesity in Asians [4]. In fact, clinical measurements of AO such as waist circumference and waist-to-hip ratio (WHR) are regarded as independent predictors for metabolic syndrome such as diabetes, hypertension, and cardiovascular diseases [5–7]. The Bangladesh study also suggested that high waist circumference and WHR reflecting AO are significant predictors of pre-diabetes [8]. Therefore, identifying AO appears to be particularly important among the Asian population at a risk of type 2 diabetes at normal BMI values [4, 9, 10]. It has been hypothesized that quantitative analysis of plasma metabolites can reveal the precise mechanisms by which or where a specific metabolic pathway is altered and deregulated in pathogenic conditions. Many studies have been conducted to identify predictive biomarkers of diabetic susceptibility. However, no biomarkers explaining the relationship between AO and progression of diabetes are yet available. Moreover, only few studies have used lipidomic analysis to characterize the relationship between AO and progression of diabetes.

Therefore, the aim of the present study was to identify AO-mediated specific biomarker candidates through the analysis of plasma lipids according to the presence or absence of abdominal obesity in normal, pre-diabetic and diabetic patients. To identify early biomarker candidates in plasma of pre-diabetic and diabetic patients with abdominal obesity, we performed a semi-quantitative lipidomic profiling using liquid chromatography-tandem mass spectrometry (LC-MS/MS) as well as routine clinical biochemical analysis of individual plasma samples. Our finding suggests that subjects who have AO may be prevented early from development of diabetes through clinical treatment because they are in the pre-diabetic stage when the serum level of SM rises.

## Materials and methods

### Study subjects and design

This study was approved by the Institutional Review Boards of Keimyung University Dongsan Medical Center in Korea (2015-03-010) and written informed consents were obtained from all subjects. We conducted surveys and clinical examination of 63 male participants who visited the Keimyung University Dongsan Medical Center for health and medical examination from May 2016 to April 2017. The subjects were divided into three groups based on fasting blood glucose and glycated hemoglobin (HbA1c) levels: patients with type 2 diabetes (DM, *n* = 25) if FBS > 126 mg/dL or HbA1c > 8.0 mmol/L, pre-diabetes (pre-DM, *n* = 18) if FBS ≥ 110±5 mg/dL or HbA1c ≥ 5.7±0.2 mmol/L, and non-diabetic control (*n* = 20) if FBS < 100 mg/dL or HbA1c < 5.7 mmol/L. The optimal cut-off of waist circumference for the criteria of abdominal obesity is 90 cm in men [11].

### Anthropometric and clinical measurements

A detailed questionnaire was completed by each of the 63 participating subjects. Information obtained included age, gender, height, weight, blood pressure, smoking history, alcohol consumption, duration of diabetes, and history of hypertension or cardiovascular diseases. All measurements were taken after an 8-h overnight fast. Trained research nurses performed blood pressure as well as anthropometric measurements using standardized protocols. Anthropometric data included height measured by using a stadiometer (FA600, Fanics, Seoul, Korea). Inbody770 (Inbody, Seoul, Korea) was used to measure body weight and body composition. BMI was calculated as weight in kilograms divided by the square of the height in meters. Waist circumference was measured midway between the lower rib margin and iliac crest. Hip circumference was measured at the level of widest circumference over the greater trochanters. WHR was calculated as waist circumference divided by hip circumference. Blood samples were obtained and used for the determination of glucose, insulin, HbA1c, alanine aminotransferase (ALT), aspartate aminotransferase (AST), triglycerides (TG), high-density lipoprotein (HDL) cholesterol, and low-density lipoprotein (LDL) cholesterol. The biochemical profiles from blood samples, were analyzed by using an automated glycohemoglobin analyzer HLC-723G8 (Tosoh, Tokyo, Japan), clinical chemistry system analyzer ADVIA2400 (SIEMENS, München, Germany), and BIOSEN C-line, clinic (EKF Diagnostic, Barleben, Germany). Plasma samples for lipidomic study were stored at -80°C.

### Reagents

Lipid standards (phosphatidylcholine (PC) 17:0/14:1, phosphatidylethanolamine (PE) 17:0/14:1, lysoPC (LPC) 17:1, lysoPE (LPE) 17:1, sphingomyelin (SM) d18:1/12:0, ceramide d18:1/12:0, diacylglycerol (DAG) 8:0/8:0, triacylglycerol 15:0/15:0/15:0, and cholesteryl ester (CE) 15:0) were obtained from Avanti Polar Lipids (Alabaster, AL, USA) or Sigma-Aldrich (St. Louis, MO, USA). Lipid internal standard (IS) solutions were stored at -80°C. Ammonium acetate, chloroform, methyl *tert*-butyl ether (MTBE), and butylated hydroxytoluene were purchased from Sigma Aldrich (St. Louis, MO, USA).

### Lipid extraction

Plasma lipids were extracted by using the Matyash method with some modifications [12]. Briefly, plasma (10 μL) was aliquoted in an Eppendorf tube containing the internal lipid standard mixture (40–400 ng/mL). Four hundred microliters of ice-cold 75% methanol with 0.1% butylated hydroxytoluene (BHT) was added to the plasma sample. Next, 1 mL MTBE was added, and the mixture was shaken for 1 h at room temperature. For phase separation, 250 μL of water was added, and centrifuged (14,000 x *g*, 4°C, 15 min). The upper phase was transferred to a new tube, dried under vacuum and reconstituted in 100 μL chloroform/methanol (1:9, *v/v*).

### LC-MS/MS

Semi-quantitative lipid profiling was performed by using a Nexera2 LC system (Shimadzu Corporation, Kyoto, Japan) connected to a triple quadrupole mass spectrometer (LC-MS 8040; Shimadzu, Kyoto, Japan) with reversed phase Kinetex C18 column (100 × 2.1 mm, 2.6 μm, Phenomenex, Torrance, CA, USA) for chromatographic separation of lipids. The mobile phase A consisted of water/methanol (1:9, *v/v*) containing 10 mM ammonium acetate, and the mobile phase B consisted of isopropanol/methanol (5:5, *v/v*) containing 10 mM ammonium acetate. The gradient elution program was as follows: 0 min (30% B), 0–15 min (95% B), 15–20 min (95% B), and 20–25 min (30% B). The flow rate was set a 200 μL/min. Five microliters of sample were injected for each run. Quantitation was performed by selected reaction monitoring (SRM) of the [M+H]^+^ (or [M+NH_4_^+^]) ion and the related product ion for each lipid. To determine the concentration of each target lipid species, the calculated ratio of target analyte and internal standard (IS) is then multiplied by the concentration of the IS [13–15]. An IS for each lipid class were selected for single-point calibrations of each target lipid species (LPC 17:1, LPC 17:1, LPE 17:1, PC 31:1(17:0/14:1), PC 31:1(17:0/14:1), PC 31:1(17:0/14:1), PE 31:1(17:0/14:1), PE 31:1(17:0/14:1), SM 30:1(d18:1/12:0), ceramide 15:1, CE 15:0, DAG 16:0(8:0/8:0), and TAG 45:0(15:0/15:0/15:0) for LPC, plasmenyl LPC, LPE, PC, plasmenyl PC, plasmanyl PC, PE, plasmenyl PE, SM, ceramide, CE, DAG, and TAG class, respectively). The SRM transitions and collision energies determined for each lipid are listed in S1 Table. The total ion chromatogram (A) and SRM chromatogram (B) indicating retention time ranges for each lipid species are represented in S1 Fig.

### Statistical analysis

Statistical analyses were performed by using SPSS v.23.0 (IBM SPSS Statistics 23, Chicago, IL, USA). The results were expressed as the mean ± standard error (SE). Statistical significance was considered with a two-tailed *p*-value of < 0.05. The statistical comparison was conducted by using Student’s independent t-tests or Mann–Whitney U-test to compare continuous data between lean and AO groups. Differences among three groups (Normal, pre-DM, and DM) were determined by analysis of variance (ANOVA) followed by Tukey’s honest significant difference test. Spearman's rank correlation coefficient analysis was carried out to explore correlations between the clinical parameters and the lipid profiles. Only significant correlations were reported in the figures. Correlation matrixes were created to visualize the correlation between biochemical parameters and plasma lipids of the study participants using R (R vision 3.1.2, R Foundation for Statistical Computing, Vienna, Austria).

## Results

### Clinical characteristics of the study participants

Abdominal obese participants from all three groups had significantly higher anthropometric measurements than lean ones did, namely body weight, BMI, body fat mass, body fat%, waist circumference, and WHR. ALT and AST were significantly elevated in pre-diabetic subjects with AO (*p* = 0.001 and *p* = 0.007, respectively) compared to pre-diabetic subjects without AO (Table 1).

**Table 1:** Clinical characteristics of the study participants.

	Normal			Pre-diabetes			Diabetes		
	Lean (*n* = 10)	AO (*n* = 10)	p-value	Lean (*n* = 8)	AO (*n* = 10)	p-value	Lean (*n* = 12)	AO (*n* = 13)	p-value
**Age (year)**	39.9±1.0	42.4 ±2.4	0.347	44.9 ±2.0	43.9±1.1	0.660	51.7 ±1.9	49.1 ±2.4	0.583
**Height (cm)**	173.8±1.7	174.9 ±1.4	0.629	170.4 ±1.6	175.0±1.7	0.071	168.7 ±1.7	175.0 ±1.8	**0.016**
**Weight (kg)**	62.1±1.4	75.0 ±1.4	**0.001**	70.8 ±2.6	80.2±1.9	**0.009**	67.3 ±1.9	84.8 ±2.6	**0.001**
**BMI (kg/m2)**	21.1±0.3	24.4 ±0.2	**0.001**	24.6 ±0.5	26.3±0.5	**0.025**	23.8 ±0.4	27.7 ±0.7	**0.001**
**Soft Lean Mass(kg)**	51.4±1.5	52.4 ±1.2	0.436	53.4 ±1.8	57.4±1.6	0.116	51.9 ±2.1	55.4 ±3.4	0.388
**Fat Free Mass (kg)**	54.3±1.6	55.5 ±1.3	0.393	56.4 ±1.9	60.8±1.7	0.115	54.9 ±2.3	58.5 ±3.5	0.393
**Skeletal Muscle Mass(kg)**	30.6±1.0	31.2 ±0.8	0.606	31.8 ±1.1	34.5±1.0	0.098	30.5 ±1.2	42.8 ±10.1	0.250
**Body Fat Mass (kg)**	9.3±0.5	19.3 ±0.6	**0.001**	15.0 ±1.0	19.7±0.8	**0.002**	14.6 ±1.0	34.0 ±11.5	0.115
**Body fat (%)**	14.8±1.0	25.9 ±0.8	**0.001**	21.0 ±0.9	24.5±1.0	**0.021**	21.2 ±1.3	27.9 ±1.9	**0.010**
**Waist (cm)**	76.4±0.6	92.1 ±0.7	**0.001**	84.8 ±1.1	92.8±0.8	**0.001**	84.2 ±1.2	97.0 ±1.8	**0.001**
**Hip (cm)**	92.7±0.6	98.1 ±0.5	**0.001**	97.5 ±1.3	101.6±0.9	**0.018**	96.2 ±1.2	100.0 ±1.6	0.068
**WHR**	0.82±0.01	0.94 ±0.01	**0.001**	0.87 ±0.01	0.92±0.01	**0.004**	0.88 ±0.01	0.97 ±0.02	**0.001**
**HbA1c (%)**	5.12±0.08	5.25 ±0.1	0.247	5.86 ±0.1	5.96±0.05	0.234	7.91 ±0.41	8.60 ±0.46	0.275
**FBS (mg/dL)**	85.0±2.0	92.3 ±1.7	**0.011**	105.1 ±2.3	106.2±1.7	0.829	133.7 ±11.9	154.4 ±9.9	0.076
**ALT (IU/L)**	15.2±2.5	38.8 ±13.8	0.075	16.3 ±2.4	34.4±4.4	**0.001**	22.4 ±4.1	51.4 ±15.0	0.083
**AST (IU/L)**	19.6±1.2	28.3 ±5.4	0.393	18.1 ±1.2	24.9±1.8	**0.007**	23.4 ±2.3	36.8 ±7.2	0.280
**Cholesterol (mg/dL)**	178.5±5.9	208.5 ±8.3	**0.009**	219.4±13.9	220.6±8.6	0.939	164.2 ±13.8	180.4 ±14.8	0.442
**TG (mg/dL)**	99.1±12.7	175.6 ±24.5	**0.015**	197.8 ±53.0	240.1±41.6	0.360	171.5 ±27.1	232.8 ±54.4	0.334
**HDL (mg/dL)**	53.7±3.7	45.7 ±3.2	0.121	45.3 ±1.9	41.3±2.3	0.274	41.7 ±2.7	44.2 ±3.8	0.650
**LDL (mg/dL)**	103.7±7.5	128.5 ±6.8	**0.025**	139.6 ±10.4	137.0±8.0	0.848	91.4 ±10.7	97.8 ±10.4	0.675
**Creatine (mg/dL)**	0.88±0.02	0.9 ±0.03	0.853	0.88 ±0.06	0.91±0.03	0.594	0.92 ±0.06	0.85 ±0.04	0.318

Values are given as mean ± SE. Significant differences between lean subjects and subjects with abdominal obesity (AO) were analyzed by Student’s t-test or Mann-Whitney. Bold font indicates significance at *p* < 0.05. Abbreviations: BMI, body mass index; WHR, waist to hip ratio; HbA1c, hemoglobin A1c; FBS, fasting blood sugar; ALT, alanine aminotransferase; AST, aspartate aminotransferase; TG, triglyceride; HDL, high-density lipoprotein cholesterol; LDL, low-density lipoprotein cholesterol.

### Identification of lipids in the human plasma using LC-MS/MS

In general, reversed-phase LC (RPLC), normal-phase (NPLC) and hydrophilic interaction chromatography (HILIC) method have been used for the analysis of lipid mixtures [16]. Among them RPLC has been most widely used for the analysis of complex lipids in various biological samples [16]. In addition, the analysis time in NPLC and HILIC are typically longer (30–60 min) than RPLC [16]. Therefore, we used RPLC method for the analysis of plasma lipids in this study [17].

The mass spectrometry method was based on the automated acquisition of 204 SRM transitions (S1 Table). Specific diagnostic Q1 and Q3 ions were used to quantify each lipid class. [M+NH_4_] ^+^ ion for neutral lipids, including DAG, TAG, and CE were selected as Q1 ion, whereas [M+H] ^+^ ion for the other lipids were selected. Lipid class specific ions or fragment ion produced from the loss of fatty acyl chain were selected as Q3 ion for the quantitation of each lipid. For example, five PC species (LPC, PC, plasmanyl PC, plasmenyl PC, and SM), two LPC species (plasmanyl LPC and plasmenyl LPC), CE, and ceramide were recorded by Q3 ion at *m/z* 184, 104, 369, and 264 corresponding to phosphocholine, choline, cholesterol-H_2_O, and sphingosine-2H_2_O, respectively [18, 19]. The fragment ions from the loss of the phosphoethanolamine group (-141) and fatty acyl chain were selected as Q3 ion for PE (LPE and PE) and neutral lipids (DAG and TAG), respectively [20]. For LPC, PC, plasmanyl PC, plasmenyl PC, and SM species, the transition from the protonated molecular ion ([M+H]^+^) to the polar head fragment ion (*m/z* 184, phosphocholine) was recorded for the quantitation [21]. Regarding DAG and TAG species, the transition from the ammoniated molecular ion ([M+NH_4_] ^+^) to the fragment ions from the loss of fatty acyl chain were recorded (S1 Fig).

### Effect of abdominal obesity on plasma lipid levels

**Fig 1:**
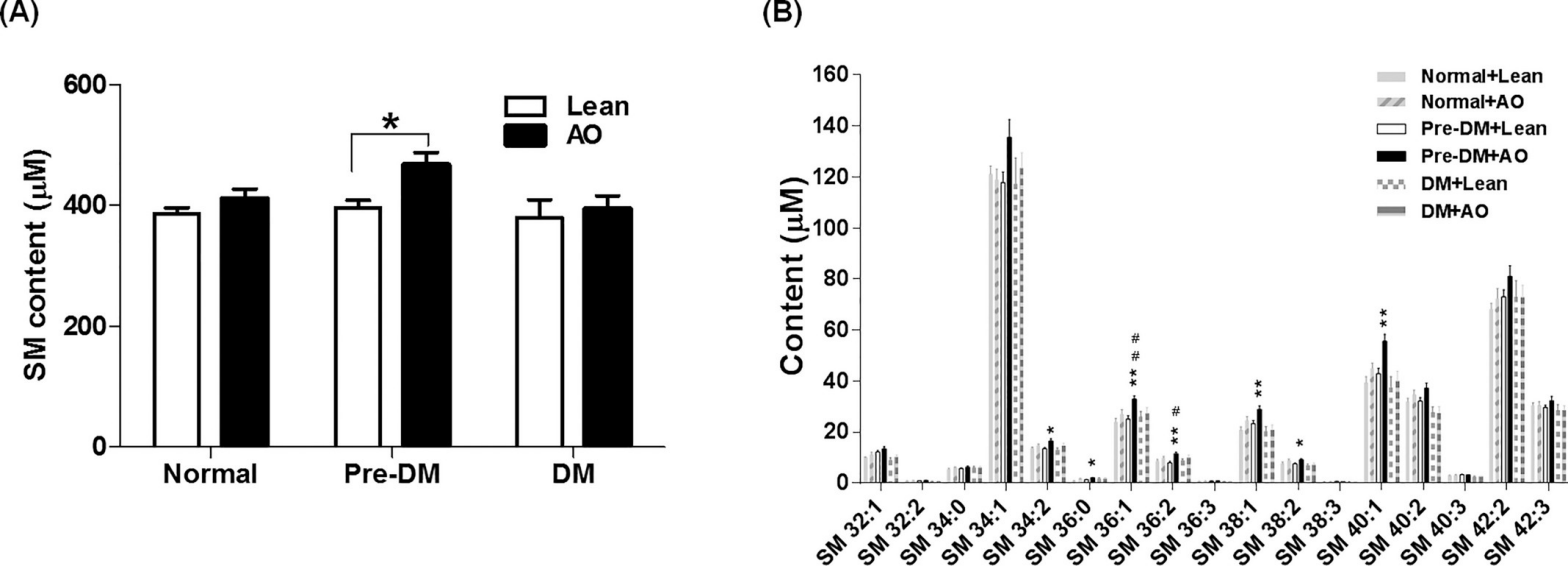
Changes in sphingomyelin (SM) content. (A) Plasma concentration of total SM in lean subjects and abdominal obese (AO) subjects in the normal, pre-diabetes (Pre-DM), and diabetes (DM) groups. Plasma SM species were determined by liquid chromatography-tandem mass spectrometry, and total SM content was obtained by the sum of each SM concentration. Data are expressed as the mean ± SE. *P*-values were derived from an independent *t*-test (lean *vs*. AO, * *p* < 0.05) and from a one-way analysis of variance (ANOVA) with Tukey's post hoc tests between the groups with AO (^#^ *p* < 0.05). (B) Content of each SM species between groups. Profiles of each SM species with carbon numbers from 32−44 in human blood plasma were determined by liquid chromatography-tandem mass spectrometry. Bars represent mean ± SE. Significant differences between lean and AO groups are indicated by * *p* < 0.05, ** *p* < 0.01. Significant differences between Normal and Pre-DM in AO groups are indicated by # *p* < 0.05, ## *p* < 0.01.

Of the 12 lipid classes, the levels of total TAG were significantly elevated by AO in normal subjects (*p* = 0.025), whereas the levels of the other lipids were not significantly altered by AO (Table 2). The levels of total SM were significantly increased in the pre-diabetic subjects with AO compared to the pre-diabetic subjects without AO (*p* = 0.019) (Fig 1A). Otherwise, the other lipids were not significantly altered by AO (Table 2). We further examined the change of 17 individual SM species among the six groups. Higher concentrations (> 20 μM) of SM 34:1, 36:1, 38:1, 40:1, 40:2, 42:2, and 42:3 were found in the plasma. Seven SM species (34:2, 36:0, 36:1, 36:2, 38:1, 38:2, and 40:1) were significantly increased in pre-diabetic subjects with AO compared with the pre-diabetic subjects without AO (Fig 1B). In addition, Pre-DM patients with AO had significantly higher levels of SM36:1, SM38:1 and SM40:1 than normal subjects with AO (Table 3). Taken together, these data suggest that SM36:1, SM38:1 and SM40:1 among lipid species might be putative diagnostic markers in pre-diabetic patients with AO.

**Table 2:** Plasma lipid profiles.

	Normal			Pre-diabetes			Diabetes		
Lipid (μM)	Lean (*n* = 10)	AO (*n* = 10)	*p*-value	Lean (*n* = 8)	AO (*n* = 10)	*p*-value	Lean (*n* = 12)	AO (*n* = 13)	*p*-value
**TAG**	314.1±38.6	526.1±74.5	**0.025**	593.2±133.9	777.3±129.0	0.341	627.0±95.8	616.±109.8	0.644
**DAG**	9.8±1.3	14.0±2.1	0.105	17.5±3.5	17.9±2.9	0.917	25.9±6.4	26.9±7.5	0.605
**SM**	386.7±9.7	410.8±16.4	0.221	396.3±12.3	467.0±20.7	**0.019**	380.1±29.5	393.8±22.3	0.711
**Ceramide**	2.82±0.2	3.1±0.2	0.448	3.6±0.3	3.8±0.3	0.460	2.6±0.4	2.4±0.2	0.689
**CE**	14957.1±438.6	15609.7±618.9	0.401	16905.1±602.9	15791.3±514.8	0.177	10308.6±1394.6	10877.7±1318.5	0.786
**PC**	1408.8±37.2	1503.2±74.9	0.165	1633.9±124.9	1772.0±109.8	0.418	1509.1±102.2	1640.0±108.3	0.390
**LPC**	331.6±12.8	318.6±15.7	0.531	321.8±29.3	293.4±21.8	0.439	247.5±25.6	224.2±20.5	0.482
**Plasmenyl PC**	27.7±0.8	30.0±2.7	0.971	30.8±1.53	29.0±1.5	0.438	26.1±1.8	27.3±2.3	0.668
**Plasmanyl PC**	142.9±2.2	154.9±7.3	0.105	168.5±8.0	178.7±8.8	0.418	140.0±7.2	154.2±10.4	0.282
**PE**	15.2±1.2	13.5±1.5	0.377	18.6±4.0	21.4±4.7	0.657	20.3±2.4	23.5±3.2	0.442
**LPE**	9.3±0.6	8.1±0.7	0.213	9.2±1.9	7.8±0.9	0.689	7.2±0.8	7.0±0.5	0.792
**Plasmenyl PE**	12.1±1.1	15.9±1.8	0.190	16.4±1.5	14.1±1.4	0.259	17.1±3.1	18.3±3.2	0.978

Values are given as mean ± SE. Significant differences between lean and abdominal obesity groups were analyzed by Student’s t-test or Mann-Whitney. Bold font indicates significance at *p* < 0.05. Abbreviations: TAG, triacylglycerol; DAG, diacylglycerol; SM, sphingomyelin; CE, cholesteryl esters; PC, phosphatidylcholine; LPC, lysophosphatidylcholine; Plasmenyl PC, plasmenyl phosphatidylcholine; plasmanyl PC, plasmanyl phosphatidylcholine; PE, phosphatidylethanolamine; LPE, lysophosphatidylethanolamine; Plasmanyl PE, plasmenyl phosphatidylethanolamine.

**Table 3:** Sphingomyelin species profiles.

	Normal			Pre-diabetes			Diabetes			*p*-value^b^ (Normal+AO vs. Pre-DM+AO)
SM (μM)	Lean (n = 10)	AO (n = 10)	*p*-value^a^	Lean (n = 8)	AO (n = 10)	*p*-value^a^	Lean (n = 12)	AO (n = 13)	*p*-value^a^	
**SM 32:1**	9.9±0.18	11.1±0.87	0.353	12.0±0.67	13.3±0.89	0.297	9.0±0.92	10.1±0.81	0.225	0.095
**SM 32:2**	0.82±0.05	0.95±0.06	0.105	0.83±0.05	0.98±0.05	0.050	0.63±0.05	0.67±0.07	0.622	0.698
**SM 34:0**	5.4±0.24	6.0±0.27	0.142	5.6±0.30	6.1±0.46	0.740	6.0±0.52	6.0±0.51	0.971	0.825
**SM 34:1**	121.1±3.11	119.0±4.09	0.681	117.8±4.10	135.6±6.91	0.067	117.3±10.23	123.5±6.04	0.604	0.053
**SM 34:2**	13.6±0.42	14.8±0.47	0.069	13.5±0.36	16.5±0.91	**0.019**	13.0±0.68	14.67±0.79	0.135	0.127
**SM 36:0**	0.83±0.09	1.67±0.14	**0.001**	1.23±0.16	1.94±0.23	**0.037**	1.68±0.24	1.74±0.31	0.871	0.336
**SM 36:1**	24.0±1.43	27.1±1.53	0.165	25.1±1.32	32.9±1.25	**0.001**	26.0±2.1	27.3±2.31	0.687	**0.009**
**SM 36:2**	8.7±0.55	9.9±0.52	0.111	7.9±0.48	11.5±0.61	**0.001**	8.8±0.55	9.9±0.93	0.347	0.071
**SM 36:3**	0.63±0.04	0.66±0.05	0.643	0.61±0.03	0.76±0.05	0.088	0.55±0.13	0.56±0.05	0.793	0.191
**SM 38:1**	20.7±1.24	24.6±1.37	**0.049**	23.2±1.31	28.9±1.29	**0.009**	20.3±1.73	20.8±1.99	0.856	**0.036**
**SM 38:2**	7.7±0.52	8.8±0.47	0.105	7.4±0.50	9.1±0.45	**0.024**	7.0±0.39	7.1±0.65	0.904	0.650
**SM 38:3**	0.43±0.03	0.53±0.04	0.089	0.53±0.04	0.48±0.02	0.364	0.43±0.04	0.47±0.04	0.490	0.343
**SM 40:1**	39.4±2.3	45.0±2.0	0.080	42.9±2.08	55.5±2.70	**0.004**	37.6±4.04	39.9±3.90	0.694	**0.006**
**SM 40:2**	31.9±1.3	34.8±1.5	0.170	32.1±1.43	37.1±2.02	0.083	27.7±2.09	27.5±2.44	0.943	0.378
**SM 40:3**	2.9±0.16	3.09±0.16	0.362	3.1±0.18	3.06±0.15	0.735	2.4±0.24	2.5±0.18	0.705	0.895
**SM 42:2**	68.2±2.16	72.4±3.84	0.393	73.0±2.67	81.0±4.10	0.159	73.0±6.30	72.8±4.75	0.976	0.140
**SM 42:3**	30.6±0.80	30.4±1.51	0.899	29.6±0.92	32.3±1.66	0.229	28.5±2.29	28.5±1.74	0.994	0.423

Values are given as mean ± SE. Differences between two groups were tested by Student’s t-test or Mann-Whitney. *p* -values^a^ were used to determine statistical significance between lean and abdominal obesity groups. P-values^b^ were derived from Normal + AO vs. Pre-DM + AO. Bold font indicates significance at *p* < 0.05.

### Correlation among biochemical parameters and plasma lipids

**Fig 2:**
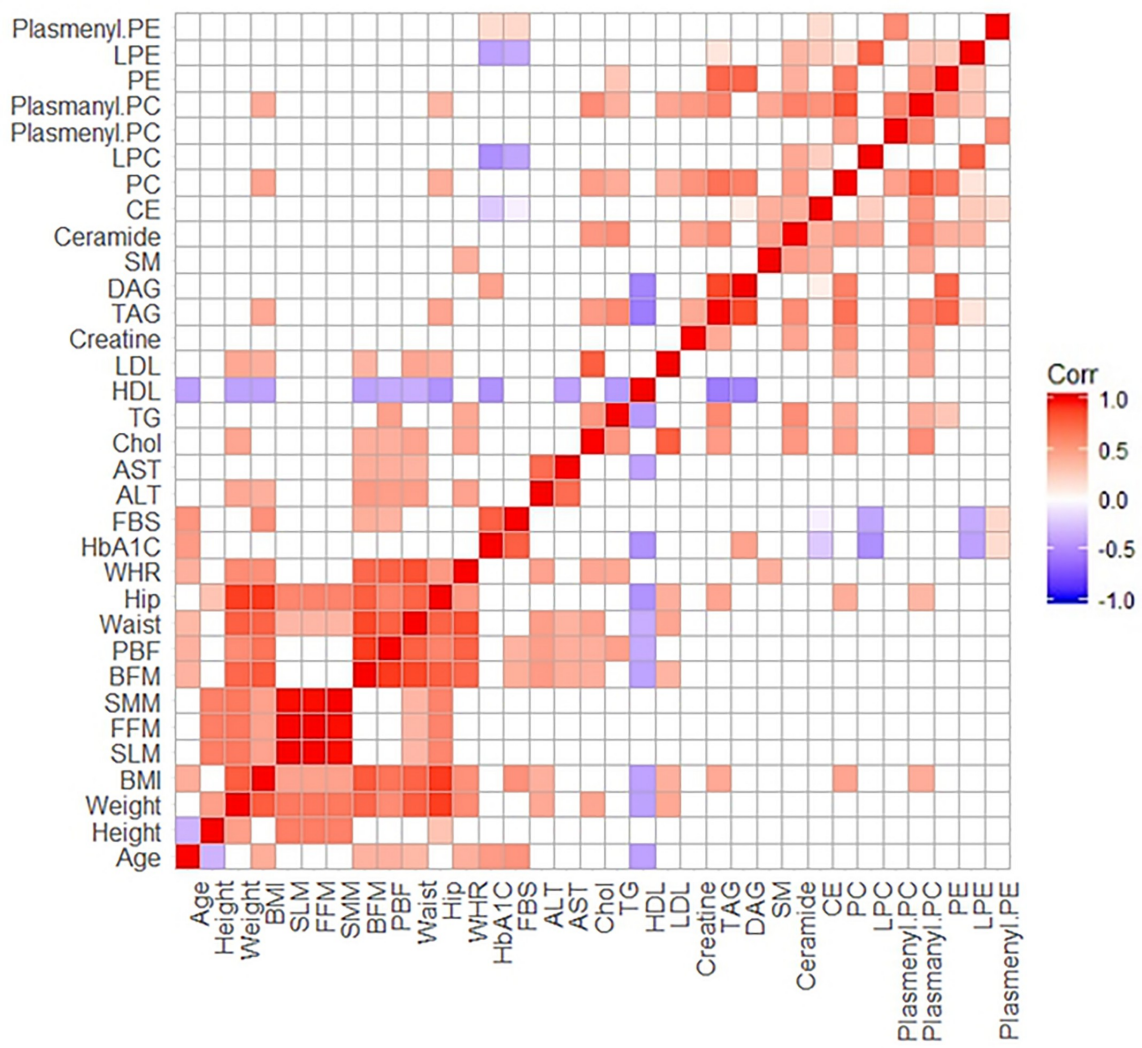
Correlation matrix for biochemical characteristics and plasma lipid metabolites across all participants. The correlations were obtained by deriving *Pearson*’s correlation coefficients. Red represents a positive correlation and blue represents a negative correlation.

**Fig 3:**
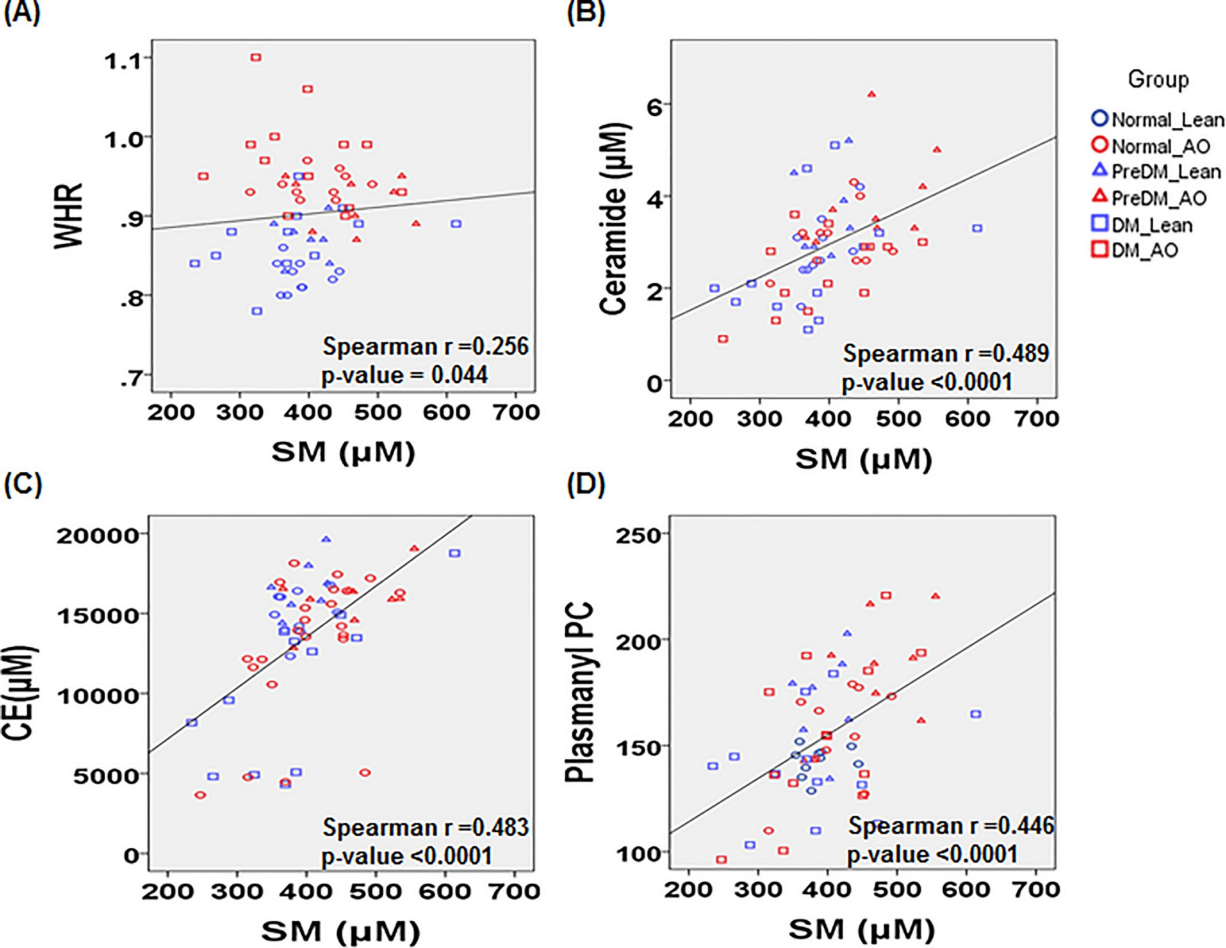
**Correlation scatter plots for total sphingomyelin (SM) level with (A) WHR, (B) CE, (C) Ceramide and (D) Plasmanyl PC in all participants.** Circles represent normal, triangles represent pre-diabetes (pre-DM) and squares represent type 2 diabetes (DM). Blue and red indicate lean and abdominal obesity (AO), respectively.

The Spearman’s correlation coefficients between the anthropometric measures and biochemical parameters with 12 lipid classes. A correlation matrix was computed for all participants (*n* = 63). Interestingly, LPC and LPE had a negatively correlation with classical diagnostic markers of diabetes or pre-diabetes like FBS and HbA1c. Total SM was positively correlated with WHR and 3 lipid molecular species, such as CE, ceramide and plasmanyl PC (Fig 2). This observation provides additional evidence that SM might be a putative mediator between abdominal obesity and pre-diabetes. Total SM showed a moderate correlation with CE (*r* = 0.483, *p* < 0.0001), ceramide (*r* = 0.489, *p* < 0.0001) and plasmanyl PC (*r* = 0.446, *p <* 0.0001), and a weak correlation with WHR (*r* = 0.256, *p* = 0.044) (Fig 3).

## Discussion

According to a recent trend of type 2 diabetes in Korea, the prevalence of diabetes is increasingly shifting to obese people, and an inverse linear relationship was detected between BMI and age at diabetes diagnosis among newly diagnosed patients [1]. Nevertheless, there are many non-obese but metabolically unhealthy individuals. Previous research demonstrated that the risk of type 2 diabetes starts at a lower BMI for Asians than for Europeans [22]. Lear *et al*. determined that healthy Chinese and South Asian individuals present with more visceral adipose tissue than Europeans with the same BMI or waist circumference [23]. Mechanistic studies revealed that visceral adiposity is strongly associated with systemic insulin resistance, which may be one of the key pathophysiological processes in the development of diabetes [2, 24, 25]. The accumulation of visceral and omental fat tissue may induce local inflammation and release various adipocytokines, leading to systemic insulin resistance [2].

In this study, we report the results of plasma lipidomic analysis of 63 participants categorized as normal, pre-diabetic, and diabetic subjects with or without AO. Interestingly, plasma SM levels (SM 36:1, SM 38:1, and SM 40:1), which are related with fatty liver and insulin resistance as well as obesity, were significantly elevated in pre-diabetic individuals with AO when compared to normal individuals with AO or pre-diabetic without AO (Fig 1B and Table 3). Even though these findings were similar with previous studies reported that serum SM species (SM 36:1, 38:1, 40: 1, and 42:1) were high in the obese groups [26, 27], our study found that SM species level was signicantly increased in pre-diabetic patients with AO compared to pre-diabetic individuals without AO. The results suggest that the abdominal fat accumulation in Korean patient might enhance increase of SM secretion uniquely in pre-diabetic patient than in diabetic patients.

SM is one of the abundant lipids in sphingolipid species and plays major functional roles in animal plasmalemma and membranes of cellular organelles as well as a membrane lipid component *per se* [28, 29]. SM has a higher concentration than ceramide, which means that SM may act as a pool for the rapid generation of ceramide due to SM hydrolysis by sphingomyelinases. The cited authors also found that high-fat diet feeding significantly increased SM level in the blood and various tissues such as the liver, adipose tissue and heart [30–34]. Consistently, reducing plasma SM levels increased insulin sensitivity in sphingomyelin synthase 2 (Sms2) knockout mice [35]. Taken together, these findings suggest that SM can induce systemic insulin resistance and thus glucose intolerance. The present findings further suggest that increased visceral fat accumulation may be a cause of increased plasma SM levels. Therefore, the detection of high plasma levels of SM in individuals with AO may be an early sign of pre-diabetes and may be a biomarker of diabetes development [36]. General or systemic obesity determined by BMI and total body fat mass is known as a sign for insulin resistance in the early progression of diabetes [37]. In addition, we hypothesize that local omental or visceral fat accumulation with increased plasma SM levels might be also a biomarker of pre-diabetes. As all lipids are not equally affected by obesity, further studies are warranted to determine the mechanism underlying the increase in SM and how SM induces glucose intolerance in subjects with diabetes with AO [38–40]. The link between SM and diabetes has not been clearly elucidated, but previous data have shown that clearance synthesis and apoptosis in apoE knockout mice result in increased SM content in lipoproteins [41]. As the SM content of slowly removed residual lipoproteins increases, the susceptibility to arterial wall sphingomyelinase, which plays an important role in converting the sphingomyelin into ceramide, is decreased, leading to an increase in plasma SM level and increase of incidence of atherosclerosis.

In the pre-DM group, AO was also associated with higher plasma AST and ALT levels, reflecting fatty liver and hepatic lipotoxicity. AST and ALT levels correlated positively with obesity-related parameters such as body fat mass and percentage body fat as well as with AO-related waist circumference. Increased visceral fat, having greater lipolytic potential, may result in increased delivery of free fatty acid to the liver, which may in turn lead to fatty liver, hepatic insulin resistance and liver damage [42, 43].

In conclusion, the data demonstrated a greater difference in SM species in pre-diabetic subjects with AO. SM levels in the plasma may be an important predictor of early diabetes in Korean men with AO and might be useful to monitor disease progression. Under abdominal obesity condition, identifying pre-diabetes may help develop preventative strategies or better therapeutic approaches based on specific pathogenic mechanisms [44]. This finding should be confirmed in prospective studies with a larger sample size and a multicenter study. Further study is required at a molecular level to figure out an underlying mechanism of lipid metabolites and their enzymes.

## Supporting information

S1 Table The SRM transitions and collision energies determined for each lipid. (XLSX)

S1 Fig The total ion chromatogram (A) and SRM chromatogram (B) of various lipids in human plasma.
(TIF)
