# Supplementary figures and images for: Plasma sphingomyelins increase in pre-diabetic Korean men with abdominal obesity
Source: PLoS One. 2019 Mar 5;14(3):e0213285. doi: 10.1371/journal.pone.0213285 (PMC6400388; doi:10.1371/journal.pone.0213285)

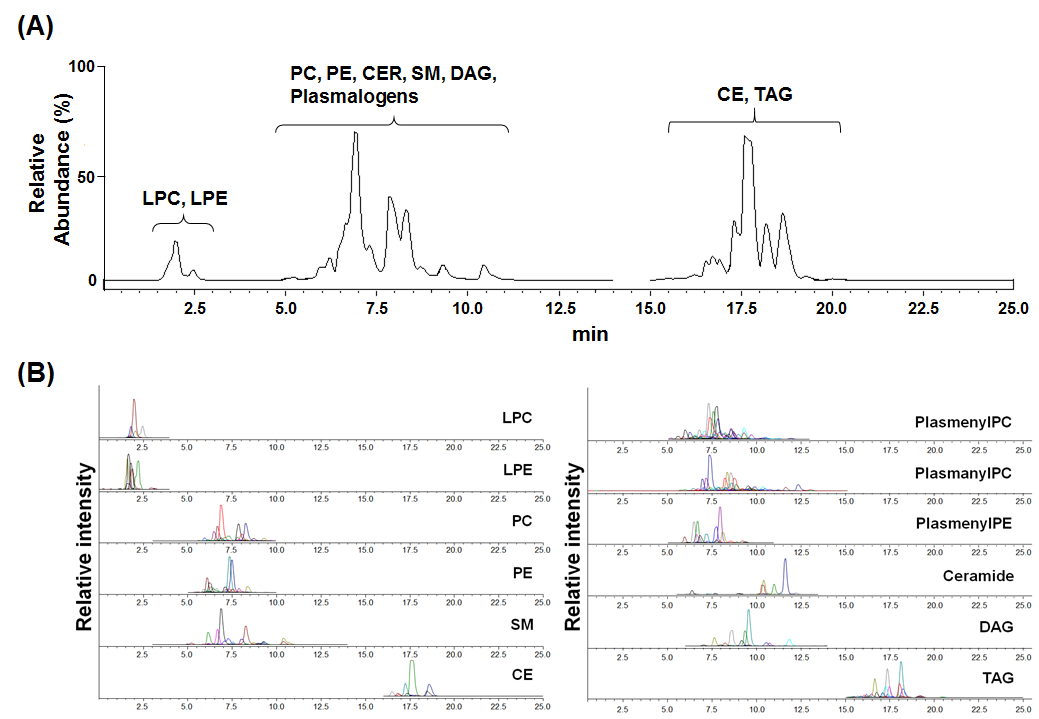

Supplement: S1 Fig — The total ion chromatogram (A) and SRM chromatogram (B) of various lipids in human plasma. (TIF) [file pone.0213285.s002.tif]
